# Confirmatory assays are essential when using molecular testing for *Neisseria gonorrhoeae* in low-prevalence settings: insights from the third National Survey of Sexual Attitudes and Lifestyles (Natsal-3)

**DOI:** 10.1136/sextrans-2014-051850

**Published:** 2014-12-15

**Authors:** Nigel Field, Soazig Clifton, Sarah Alexander, Catherine A Ison, Gwenda Hughes, Simon Beddows, Clare Tanton, Kate Soldan, Filomeno Coelho da Silva, Catherine H Mercer, Kaye Wellings, Anne M Johnson, Pam Sonnenberg

**Affiliations:** 1 Research Department of Infection & Population Health, University College London, Mortimer Market Centre, London, UK; 2 NatCen Social Research, London, UK; 3 Sexually Transmitted Bacteria Reference Unit, Public Health England, London, UK; 4 Public Health England, National Centre for Infectious Disease Surveillance and Control, London, UK; 5 Virus Reference Department, Public Health England, London, UK; 6 Department of Social and Environmental Research, London School of Hygiene & Tropical Medicine, London, UK

**Keywords:** NEISSERIA GONORRHOEA, TESTING, EPIDEMIOLOGY (CLINICAL), PUBLIC HEALTH

## Abstract

**Objectives:**

To investigate the occurrence of unconfirmed positive gonorrhoea results when using molecular testing within a large population-based survey.

**Design, setting and participants:**

Between 2010 and 2012, we did a probability sample survey of 15 162 men and women aged 16–74 years in Britain. Urine from participants aged 16–44 years reporting ≥1 lifetime sexual partner was tested for *Neisseria gonorrhoeae* and *Chlamydia trachomatis* using the Aptima Combo 2 (AC2) assay, with positive or equivocal results confirmed with molecular assays using different nucleic acid targets.

**Results:**

A total of 4550 participants aged 16–44 years had urine test results (1885 men; 2665 women). For gonorrhoea, 18 samples initially tested positive and eight were equivocal. Only five out of 26 confirmed, giving a positive predictive value (PPV) for the initial testing of 19% (95% CI 4% to 34%). Most (86% (18/21)) participants with unconfirmed positive results for gonorrhoea reported zero or one sexual partner without condoms in the past year and none had chlamydia co-infection, whereas all five with confirmed gonorrhoea reported at least two recent sexual partners without condoms, and four had chlamydia co-infection*.* The weighted prevalence for gonorrhoea positivity fell from 0.4% (0.3% to 0.7%) after initial screening to <0.1% (0.0% to 0.1%) after confirmatory testing. By comparison, 103 samples tested positive or equivocal for chlamydia and 98 were confirmed (PPV=95% (91% to 99%)).

**Conclusions:**

We highlight the low PPV for gonorrhoea of an unconfirmed reactive test when deploying molecular testing in a low-prevalence population. Failure to undertake confirmatory testing in low-prevalence settings may lead to inappropriate diagnoses, unnecessary treatment and overestimation of population prevalence.

## Introduction

Dual nucleic acid amplification tests (NAATs) allow simultaneous detection of *Neisseria gonorrhoeae* and *Chlamydia trachomatis*.^1^ While advantageous in some settings, the appropriate deployment of dual NAATs is complicated by the different epidemiological characteristics of these infections.^2^

National surveillance data for gonorrhoea diagnoses in specialist sexual health clinics (genitourinary medicine (GUM) clinics) show that gonorrhoea is highly concentrated geographically and in specific population groups in England (eg, men who have sex with men and black Caribbeans).^3^ Even so, gonorrhoea prevalence is below 1% in around one-third of GUM clinics (Town, Public Health England, unpublished data). Studies in community-based settings suggest that gonorrhoea prevalence ranges from 0.3% to 1.7% outside London, and up to 4.1% in South London, but might overestimate prevalence due to a lack of confirmatory testing and/or selection bias in the population tested.^4^

Using data from the third National Survey of Sexual Attitudes and Lifestyles (Natsal-3), a representative probability sample of British residents, we have previously shown that chlamydia prevalence in the sexually active general population was 1.3% in men and 1.5% in women aged 16–44 years, with half of chlamydia infections found in those with only one recent partner.^5^ By contrast, gonorrhoea prevalence was below 0.1%, with infection restricted to individuals reporting risky sexual behaviour.^5^ Since the positive predictive value (PPV) for any test decreases with lower prevalence, testing in low prevalence areas is likely to lead to an increased proportion of positive tests that do not confirm.^6^

To inform gonorrhoea testing policy, we report the occurrence of unconfirmed positive gonorrhoea results using a dual assay, uniquely linking laboratory findings with demographic and behavioural data in the British sexually active population.

## Methods

### Participants and survey procedures

Natsal-3 was a stratified probability sample survey of 15 162 men and women aged 16–74 years in Britain who were interviewed during 2010–2012. The estimated overall response rate was 57.7% and the cooperation rate was 65.8% (of all eligible addresses contacted). Participants were interviewed using computer-assisted face-to-face and self-completion questionnaires; further methodological details have been described elsewhere.^5^ After the interview, we invited a sample of participants aged 16–44 years to provide urine for sexually transmitted infection (STI) testing.^5^ The first 4–5 mL of voided urine was collected with the FirstBurst device, and posted to Public Health England for testing.

### Laboratory methods

Details of urine sample preparation, testing and quality assurance are available elsewhere.^5^ Our pre-defined testing strategy aimed to reduce the likelihood of false positives in the final prevalence estimates. A primary screening test for *C. trachomatis* and *N. gonorrhoeae* was performed using the Aptima Combo 2 (AC2) assay (Hologic Gen-Probe) using the Panther platform. Positive, equivocal or negative results were designated according to the manufacturers’ instructions. All samples that generated a positive or equivocal result (hereafter referred to as ‘reactive’) were re-tested with a secondary Aptima monospecific assay for single detection of either *C. trachomatis* (Aptima CT) or *N. gonorrhoeae* (Aptima GC). Only samples that were reactive with two tests were considered true positives for our initial paper.^5^ However, for gonorrhoea, to explore the possibility that true infections might be missed (eg, due to the high mutability of the *N. gonorrhoeae* genome leading to loss of assay targets), all specimens that generated reactive results using the AC2 test were also tested using a *N. gonorrhoeae*-specific multiplex real-time PCR as a further confirmatory assay. The multiplex real-time PCR, which targets the *opa* gene and the *porA* pseudogene using the Rotorgene 5000 platform (Qiagen)^7^ was performed using DNA extracted from a separate aliquot for each sample. For this study, we deemed a ‘confirmed’ infection to be one where the initial AC2 test was reactive and any supplementary test was positive. We used an in-house Luminex-based genotyping assay to detect human papilloma virus (HPV) types, defining high-risk (HR-HPV) types as previously described.^5^

### Statistical analysis

Survey analyses were done in Stata V.13 accounting for sample stratification, clustering and weighting. Analyses were additionally weighted for unequal urine selection probabilities and differential urine sample response.^5^

## Results

### Laboratory findings

STI test results were available from 4550 participants (2665 women; 1885 men).^5^ For gonorrhoea, the weighted prevalence for a reactive test result (18 positive; 8 equivocal), using the AC2 test, was 0.4% (95% CI 0.3% to 0.7%). No samples with an equivocal result could be confirmed with supplementary testing. Of the 18 samples with a positive result, four tested positive using the AGC test, and these samples also tested positive for the *opa* gene and *porA* pseudogene. A fifth sample tested negative using the AGC assay, but positive for *opa* and *porA*. We deemed all five samples to have confirmed gonorrhoea. The PPV for a reactive dual NAAT test was 19% (4% to 34%) among those aged 16–44 years and 29% (5% to 53%) among those aged 16–24 years. Overall, the weighted prevalence for confirmed gonorrhoea was <0.1% (0.0% to 0.1%).

By comparison, 103 samples were reactive for chlamydia using the AC2 assay (three were equivocal), of which 98 confirmed (two were initially equivocal using the AC2 assay). The PPV for chlamydia was 95% (91% to 99%). Among those aged 16–44 years, the weighted prevalence for a reactive test for chlamydia was 1.3% (1.1% to 1.8%), and not different from confirmed chlamydia (1.3% (1.0% to 1.6%)).

### Epidemiological findings

Of the five samples with confirmed gonorrhoea, two were from men aged 20–34 years and three were from women aged 20–24 years (table 1). Of the 21 participants with unconfirmed positive gonorrhoea, 67% (47% to 87%) were women and 57% (36% to 87%) were under 25 years. All five participants with confirmed gonorrhoea reported sex with two or more partners in the past year without condoms, whereas 86% (71% to 100%) of those with unconfirmed gonorrhoea reported zero or one such partner. Two men and two women with confirmed gonorrhoea were co-infected with chlamydia, and all five participants with confirmed gonorrhoea had at least one HPV type. None of the participants with unconfirmed positive gonorrhoea was co-infected with chlamydia.

**Table 1:** Prevalence of unconfirmed positive and confirmed positive *Neisseria gonorrhoeae* in participants aged 16–44 years, by sex

	Men							Women						
	Unconfirmed Positive*			Confirmed Positive†			Denominator‡	Unconfirmed Positive*			Confirmed Positive†			Denominator‡
	n	Per cent	95% CI	n	Per cent	95% CI	Unweighted, weighted	n	Per cent	95% CI	n	Per cent	95% CI	Unweighted, weighted
All	7	0.3	(0.1 to 0.6)	2	<0.1	(0.0 to 0.1)	1885, 2266	14	0.5	(0.3 to 1.0)	3	<0.1	(0.0 to 0.1)	2665, 2284
Age group														
16–19	3	0.9	(0.3 to 3.0)	0	0.0	–	343, 234	1	0.2	(0.0 to 1.4)	0	0.0	–	395, 214
20–24	1	0.1	(0.0 to 0.8)	1	0.1	(0.0 to 0.6)	497, 391	5	0.8	(0.3 to 2.2)	3	0.2	(0.1 to 0.7)	597, 383
25–34	3	0.4	(0.1 to 1.3)	1	0.1	(0.0 to 0.4)	693, 807	5	0.4	(0.2 to 1.1)	0	0.0	–	1146, 809
35–44	0	0.0	–	0	0.0	–	352, 835	3	0.5	(0.2 to 1.8)	0	0.0	–	527, 878
No. of partners without a condom, past year														
0	3	0.5	(0.1 to 1.5)	0	0.0	–	425, 494	2	0.2	(0.0 to 0.8)	0	0.0	–	469, 428
1	3	0.1	(0.0 to 0.4)	0	0.0	–	1111, 1486	10	0.6	(0.3 to 1.3)	0	0.0	–	1806, 1629
2+	1	0.6	(0.1 to 4.0)	2	0.3	(0.1 to 1.2)	322, 264	2	0.4	(0.1 to 1.7)	3	0.4	(0.0 to 0.1)	355, 200
Co-infection in urine														
Chlamydia (men & women)§	0	0.0	–	4	1.9	(0.7 to 5.1)	98, 58							
HR-HPV	0	0.0	–	1	0.2	(0.0 to 0.6)	164, 183	2	0.4	(0.1 to 1.6)	3	0.2	(0.0 to 1.2)	527, 348
Any HPV	2	0.4	(0.1 to 1.9)	2	0.2	(0.1 to 0.9)	323, 354	5	0.6	(0.2 to 1.8)	3	0.1	(0.0 to 0.4)	959, 673
Experienced urethral symptoms, past month														
No	7	0.3	(0.1 to 0.6)	1	<0.1	(0.0 to 0.2)	1781, 2160	12	0.5	(0.2 to 1.0)	3	<0.1	(0.0 to 0.1)	2436, 2105
Yes	0	0.0	–	1	0.3	(0.0 to 2.1)	102, 101	2	0.9	(0.2 to 4.3)	0	0.0	-	223, 175
Attended sexual health clinic, past 5 years														
No	4	0.2	(0.1 to 0.6)	1	<0.1	(0.0 to 0.2)	1328, 1790	8	0.5	(0.2 to 1.1)	1	<0.1	(0.0 to 0.1)	1852, 1766
Yes	3	0.5	(0.2 to 1.6)	1	<0.1	(0.0 to 0.5)	526, 439	6	0.6	(0.3 to 1.5)	2	0.1	(0.0 to 0.5)	788, 499

*Unconfirmed positive is a sample with a positive or equivocal dual NAAT result that was negative on two supplementary tests.

†Confirmed positive is a sample with a positive or equivocal dual NAAT result that was positive on at least one of three supplementary tests.

‡Denominator is Natsal-3 participants aged 16–44 years who reported at least one partner, ever.

§For chlamydia, men and women were combined because the denominators were too small for data to be reported by gender separately.

HR-HPV, high risk human papilloma virus; NAAT, nucleic acid amplification test.

## Discussion

To our knowledge, this is the first study of its kind linking laboratory data to epidemiological characteristics for individuals with unconfirmed positive and confirmed STI results in a population-based survey. We show that most reactive screening tests for gonorrhoea failed to confirm when deploying a NAAT test in the sexually active British general population. The PPV for a reactive gonorrhoea result using the AC2 assay was 19% among those aged 16–44 years, and 29% among those aged 16–24 years, who would be eligible for screening through the English National Chlamydia Screening Programme. This is substantially below the 90% cut-off recommended for clinical testing algorithms in the 2014 UK gonorrhoea testing guidance.^8^ Among the group with unconfirmed positive gonorrhoea, we identified no chlamydia co-infection and few individuals reported recent unsafe sex, whereas all those with confirmed gonorrhoea reported characteristics associated with STI acquisition and had co-infections. Overall, the prevalence of a reactive gonorrhoea result was 0.4%, whereas the true prevalence was <0.1%. These data illustrate that gonorrhoea testing without confirmatory strategies risks substantially overestimating population prevalence in a population where prevalence is low.

There are several reasons why reactive NAATs might fail to confirm for gonorrhoea, even when using highly sensitive and specific assays. The most likely explanation is the sporadic amplification of non-specific nucleic acids, but the finding might also be due to low-load infections, cross reaction with transient non-gonococcal *Neisseria* sp present in the sample or contamination with nucleic acid from the environment.^9^ We used a primary screening test with two independent supplementary tests, each with a different nucleic acid target, to confirm gonorrhoea results. Such comprehensive laboratory testing makes it unlikely that gonorrhoea was missed for samples with reactive NAAT tests. Nevertheless, we did find one sample with a false negative result (the sample was positive with the AC2 test, negative with the AGC monospecific assay and positive using the multiplex real-time PCR). Overall, these data highlight difficulties that can arise when interpreting NAAT results.

We did not undertake supplementary tests for samples testing negative on the AC2 test for logistic and cost reasons. Although our approach replicates that used by diagnostic laboratories, using the highly sensitive AC2 assay with automated testing, it remains possible that a small number of gonorrhoea infections were missed. Furthermore, urine is not considered the optimum specimen for detecting gonorrhoea in women, and the methods used might have limited test sensitivity.^5^ However, the strengths here lie in the size and representative nature of the sample, and linking of laboratory findings to detailed demographic and sexual behaviour data.

Gonorrhoea control is rightly a priority for sexual health services.^10^ Antibiotic resistance, transmission of resistant strains and the frequent occurrence among marginalised minority groups all increase public health concern about gonorrhoea. Our findings support policies that restrict unselected gonorrhoea testing by avoiding testing in low-prevalence populations where most reactive results will be unconfirmed.^8^ Although multiplex assays represent a valuable innovation, this study raises broader issues about appropriate use of these technologies and the importance of considering the underlying epidemiology of each infection. These data provide evidence that clinical management for gonorrhoea should be based on confirmatory testing strategies (ie, NAATs with a different nucleic acid target), which are essential to avoid misdiagnoses and unnecessary treatment. Studies and surveillance undertaken without confirmation testing may substantially overestimate prevalence.
